# Scar Revision

**DOI:** 10.1155/2010/545796

**Published:** 2011-06-29

**Authors:** Hayes B. Gladstone, Daniel Berg, Michel McDonald

**Affiliations:** ^1^Division of Dermatologic Surgery, Stanford University, Stanford, CA 94305, USA; ^2^Division of Dermatologic Surgery, University of Washington, 4225 Roosevelt Way NE, 4th Floor Dermatology, Box 354697 Seattle, WA 98195, USA; ^3^Division of Cosmetic Dermatology, Vanderbilt University Medical Center, 719 Thompson Lane Suite 26300, Nashville TN 37204, USA

## 1. Introduction

Scarring is the body's natural response to a wound. Yet, this healing process can create significant functional and cosmetic problems. It impacts daily life and can create issues in self-esteem. Traditionally, the treatment of scars has been surgical. Despite meticulous surgical technique, scars still form. Revision may be in the form of excising the scar to create a “thinner” line, or the surgeon may perform a geometric broken line to create an illusion of natural creases. Lasers or mechanical dermabrasion can also be performed to minimize the scar. Lasers including the pulse dye laser has also been used intraoperatively and in the acute postoperative period to suppress scar formation. Topical treatments such as silicone may also help soften a scar. Yet the cicatrix still exists.

Other types of scars such as those from inflammatory processes including acne create a skin topography that features alternating depressions and papules. There is no single satisfactory treatment for acne scars. Because of the complexity of this scarring process, a combined approach must be undertaken. 

Historically, dermabrasion has been performed. For Fitzpatrick Skin I, II, and III, conventional ablative laser resurfacing has been used. Despite many case series, it is not clear if the carbon dioxide laser or the erbium laser alone provides a long-term significant improvement. More recently, fractionated resurfacing both nonablative and ablative have been shown to have some effect on subsets of acne scars. Subscision which manually breaks apart the acne scars is often combined with the laser treatment. Subscision can be performed with Nokor needles through small puncture sites, or more recently using a roller device that percutaneously disrupts the scar. Other techniques including punch grafting and excision may ameliorate acne scars.

On the other end of the spectrum of acne and small surgical scars are those from burns. Because of the severe trauma of a burn, large deforming contractures can occur, or large areas of denuded skin. An artificial or natural skin substitute may need to be used foremost for coverage and protection from the environment, but also to minimize scars.

Perhaps the reason that clinicians have difficulty in eradicating scars is because we do not quite understand the mechanism from a cellular and molecular basis. Breakthroughs have been made in the past 20 years, particularly from fetal surgery and the discovery that fetal skin does not scar after wounding. The treatment implications of this type of research has still not been fully realized. Yet, it is no doubt that in the future, the prevention and treatment of scars will be in the form of targeted molecular therapies including the use of stem cells.

## 2. Mechanisms

It is this contrast between healing in the womb and healing after birth that the authors Satish and Kathju discuss in their article entitled *Cellular and Molecular Characteristics of Scarless versus Fibrotic Wound Healing*. They review the biology of fetal wound healing including the role of growth factors, keratinocytes, and reepithelialization and gene expression. This article is an excellent primer for those interested in fetal wound healing. Occleston et al. build upon the understanding of the mechanisms of scarring in their comprehensive article entitled *Therapeutic Improvement of Scarring: Mechanisms of Scarless and Scar-Forming Healing and Approaches to the Discovery of New Treatments*. These authors point out that scar-free to scar-forming healing really is a continuous spectrum. They discuss preclinical examples of wound healing and how this translates to humans. From a treatment standpoint, they elucidate the mechanisms of a prophylactic scar treatment.

### 2.1. Molecular Treatment of Scars

It is has long been understood that TGF B plays an important role in wound healing and scarring in human beings. In their fine manuscript entitled *Therapies with Emerging Evidence of Efficacy: Avotermin for the Improvement of Scarring*, Bush et al. evaluate the role of TGF B3 as a potential therapeutic agent. Echoing, the previous paper, these authors emphasize the importance of prophylactic treatment after surgery in order to reduce subsequent scarring. They describe the successful Phase I/II trials of this biologic agent. However, the Phase III trials (not published here) have been disappointing, so the march goes on for the optimal prophylactic biologic agent. Nevertheless, the success of the company's early trials may shed additional light on TGF-B and could lead to future discoveries by other research groups.

### 2.2. Surgical Treatment

Despite meticulous technique, surgical scars can still distort mobile anatomic regions such as the nasal ala, the lip, and the eyelid. The conventional method to lengthen a scar and change its orientation is by transposing its limbs. In *Z-plasty Made Simple*, Aasi demonstrates her technique for demystifying *Z* plasties which can be conceptually complex particularly for trainees. She offers foolproof methods with excellent results to optimize scars that need revision after Mohs Micrographic Surgery. This technique needs to be in the armamentarium of every surgeon performing skin and soft tissue surgery.

#### 2.2.1. Surgical Treatment: Special Considerations

The reduction of scarring and skin coverage in burn patients is one of the most difficult challenges facing reconstructive surgeons. Prompt replacement of the charred skin with a functional alternative is fundamental in minimizing contractures as well as eventual cosmesis. In *Long-Term Followup of Dermal Substitution with Acellular Dermal Implant in Burns and Postburn Scar Corrections*, Juhasz et al. describe their experience in using Alloderm in 18 patients as well as reviewing the various coverage options. In their study, they used the Vancouver Scar Scale with a 50-month followup. They demonstrated very favorable results when combined with a split thickness skin graft compared to more complicated flaps.

Finally, Fabbrocini et al. provide a comprehensive review of acne scars in their paper entitled *Acne Scars: Pathogenesis, Classification and Treatment*. In a very straightforward approach, the authors explain the current theory on why acne scars occur, how to classify them into atrophic, boxcar or icepick, and then how to choose the appropriate treatment(s). The authors nicely summarize the various therapeutic options including the increasingly popular technique of needling using a roller device. They tackle this topic well and astutely point out that there are no universal guidelines for treating acne scars and that randomized controlled studies are needed as well as evaluating the all-important psychological impact of this scarring on this patient population.

As editors, we can only hope that these articles stimulate further discussion and spur new research to minimize scarring and allow these patients to live healthy and active lives without disfigurement.

*Hayes B. Gladstone* *Hayes B. Gladstone* *Daniel Berg* *Daniel Berg* *Michel McDonald* *Michel McDonald*

